# Crush the Crave: Development and Formative Evaluation of a Smartphone App for Smoking Cessation

**DOI:** 10.2196/mhealth.9011

**Published:** 2018-03-02

**Authors:** Neill B Baskerville, Laura L Struik, Darly Dash

**Affiliations:** ^1^ Propel Centre for Population Health Impact Faculty of Applied Health Sciences University of Waterloo Waterloo, ON Canada; ^2^ School of Public Health and Health Systems Faculty of Applied Health Sciences University of Waterloo Waterloo, ON Canada

**Keywords:** mobile app, smoking cessation, young adult, software design, formative feedback

## Abstract

**Background:**

Emerging evidence supports the use of smartphone apps for smoking cessation, especially in young adults given their high smoking rates and high smartphone ownership rates. Although evaluative evidence is encouraging for supporting smoking cessation, there remains a paucity of research describing the design and development processes of mobile health (mHealth) interventions.

**Objective:**

The aim of this paper was to describe the process of developing Crush the Crave (CTC), an evidence-informed app to support smoking cessation in young adults, and the results of a formative evaluation of app usage behavior, as part of a broader program of research that seeks to establish the effectiveness of the CTC app.

**Methods:**

The Spiral Technology Action Research (STAR) 5-cycle model (listen, plan, do, act, and study) was employed to guide the development, implementation, and dissemination of CTC. The approach to development and formative evaluation included focus groups with young adult smokers (n=78) across 2 phases, analysis of the content of existing apps, 2 sessions with content experts, and Google Analytics to assess user behavior during a 12-month pilot.

**Results:**

LISTEN—focus groups revealed young adult smoker preferences of (1) positive reinforcement, (2) personalization, (3) social support, (4) quit support, (5) tracking the behavior, and (6) tracking quit benefits. PLAN—informed by evidence for smoking cessation, young adult preferences and an assessment of popular cessation apps, content experts produced a mind map and a storyboard describing app content and structure. DO—focus groups with young adult smokers provided feedback on the first version of the app with opinions on content and suggestions for improvement such as providing alerts and distractions from craving. ACT—refinements were made, and app content was organized using the 4 key design components informed by principles of persuasive technology for behavior change: credibility, task support, dialogue support, and social support. CTC was launched in April 2013 and piloted from the period July 2013 to June 2014 where 1987 Android users had 18,567 sessions, resulting in 59,384 page views and 89.58% (1780/1987) of users returning within the same day to use CTC. STUDY—a pragmatic randomized controlled trial of CTC was launched in August 2014 to demonstrate that including mHealth technology as a population-based intervention can help young adult smokers to quit. The results of this phase will be presented in a subsequent publication.

**Conclusions:**

CTC is one of the first smoking cessation apps designed to meet the needs of young adult smokers. The development was informed by the inclusion of young adults in the design and the systematic application of multiple stakeholder input, scientific evidence, and theory. The STAR model approach was followed from the beginning of intervention development, which should facilitate optimization of mHealth interventions in the future.

**Trial Registration:**

ClinicalTrials.gov NCT01983150; http://clinicaltrials.gov/ct2/show/NCT01983150 (Archived by WebCite at http://www.webcitation.org/6VGyc0W0i)

## Introduction

There is a need to develop innovative smoking cessation interventions directed toward young adults aged 18 to 34 years because this age demographic maintains higher smoking prevalence rates compared with their older adult counterparts [1,2], and few smoking cessation interventions are tailored to this population [3]. One promising direction is the use of smartphone technology for enhancing smoking cessation interventions directed toward this population [4]. Smartphone ownership among both US and Canadian young adults aged 18 to 34 years is nearly ubiquitous at 92% and 94%, respectively [5].

### Background

The use of smartphone apps has become particularly popular among young adults, with evidence indicating that they are the most likely age demographic to download apps and are the most intense users of apps [6]. Smartphone apps are available at any point in time and can host complex functions, including audio and video, bidirectional communication, as well as the retrieval of additional content when there is Internet connection [6]. The features enabled by smartphones are a clear advancement over websites and SMS text messaging (short message service, SMS) cessation programs because of their high potential to boost user engagement [7], which has been consistently documented as a strong predictor of smoking cessation [8-11]. Recently, researchers have also found that young adults prefer more intense smoking cessation programming than what is currently offered via SMS text messaging-based smoking cessation interventions [12,13].

While there are dozens of smoking cessation smartphone apps, only a small minority of apps for smoking cessation adhere to the US Clinical Practice Guidelines (USCPG), which serves as the current standard in smoking cessation interventions [14,15]. Even when apps do follow the USCPG, it has been suggested that this is likely not enough to promote cessation. Several meta-analyses of websites and SMS text messaging interventions that follow the USCPG reported that their average intent-to-treat, 30-day point prevalence quit rates at 12 months post randomization were remarkably similar, ranging from 7% to 10% [11,16-18]. It has been suggested, therefore, that apps that go beyond the USCPG and incorporate behavior change theories into their content hold even greater promise to produce higher quit rates [7,19].

### Literature Review

A recent systematic review assessed which smoking cessation apps available in the app stores are informed by evidence in their design [20]. The authors found that, of the 6 evidence-informed apps identified, only 3 were still running, and only 2 were ranked among the top 50 popular apps for smoking cessation [20]. Not only is there a lack of evidence-informed apps available, there is a lack of apps that specifically target young adults. Therefore, to capitalize on the potential of smartphone technology for young adult smoking cessation, as well as to help close the gap between existing smartphone apps and what works to help young adults quit smoking, we developed and piloted Crush the Crave (CTC).

CTC is a quit smoking app that specifically targets young adults, made available for Android and iOS devices in both English and French. The features and functions incorporated into the app were informed by principles of persuasive technology for behavior change [21], as well as evidence on what works to help individuals quit smoking according to the USCPG [22]. The model of principles of persuasive technology for behavior change contains 28 persuasive system design techniques that fall under 4 categories: task support, dialogue support, social support, and credibility support [23]. Task support aims to persuade the user to complete a task by supporting their efforts, such as offering craving distractions. Dialogue support provides feedback to encourage the user toward the intended behavior, such as providing rewards. Social support aims to strengthen the persuasiveness of a software system by leveraging human interactions, such as connecting with others about the desired behavior change. Finally, credibility support includes principles on designing a system that is more credible, and therefore, more persuasive.

Informed by this model, CTC offers features that include a customized quit plan, the tracking of cravings and smoking habits, notifications of money saved and health improvements achieved, direct dial-up to telephone-based support, virtual awards that credit performance toward reaching milestones, evidence-informed credible information (eg, nicotine replacement therapy), and the ability to connect with a community of people for social support via social media (eg, Facebook). Recently, Ubhi and colleagues [24] conducted a review of 137 smoking cessation apps for the presence or absence of evidence-informed behavior change techniques, and CTC addressed 4 out of 5 behavior change strategies as compared with an average of only 1 across the 137 apps reviewed. They also assessed CTC as having an ease of use score of 95%, which was the same as the average of all apps reviewed, and 82% for user engagement compared with only 45% overall.

### Objective

Emerging evidence supports the use of smartphone apps for smoking cessation [7,25-27]. However, there remains a paucity of research describing the design and development processes of mobile health (mHealth) interventions, leaving unanswered questions about how to productively leverage apps for quitting smoking [28]. To address this gap, this paper describes the process of developing the CTC app for smoking cessation and the results of a formative evaluation of app usage behavior, as part of a broader program of research that seeks to establish the effectiveness of the CTC app.

## Methods

### Overview

CTC was developed in 2012, to which the Spiral Technology Action Research (STAR) model [29] was employed to guide development, implementation, and dissemination. The STAR model includes the following 5 iterative cycles: listen (engage with end users to identify their needs and preferences), plan (develop a plan to address needs of end users), do (implement prototype and review with end users), act (launch intervention), and study (conduct ongoing evaluation) [29]. The STAR model provides a comprehensive yet practical guide for the development and evaluation of eHealth health promotion interventions.

### Phase I: Listen

To develop the content of CTC, we engaged with young adult smokers using a focus group methodology [30] to listen and collect information on end-user needs and preferences. Four focus groups were conducted in Waterloo, Ontario, and the sessions were guided by an interview schedule. Twenty-one participants were recruited by telephone via a panel of young adult smokers and were given an information letter and provided informed consent. Participants comprised 12 males and 9 females, and 9 with high school education or less, 9 with college diplomas, and 3 with university degrees. The age range was from 19 to 29 years. Fourteen participants smoked 10 or more cigarettes per day, 6 smoked less than 10 cigarettes per day, and the last one did not report the number of daily cigarettes smoked. Focus groups were split by gender. The focus group discussion began with questions around previous quit attempts and what helped them to make a quit attempt. Questions on usage of smartphone apps for quitting smoking and preferred features in an app were asked. Finally, participants were asked to describe their thoughts and feelings concerning a smartphone app concept that was displayed to them on a screen. Questions included what was liked and disliked about the app, followed by a question on whether participants thought that a smartphone app for quitting smoking would work. Focus groups lasted 1 hour, and 2 researchers participated in each group. One researcher facilitated the session, and the other assisted and took notes. The focus groups were audio-recorded and transcribed verbatim. Two independent researchers (LS and DD) analyzed the transcripts using an inductive framework approach to thematic analysis [31]. Codes were attached to text segments that appeared to indicate important material in relation to app content, and analysis progressed in an iterative fashion between the researchers to develop a set of themes that captured the essence of the focus group discussions. To validate coding, the second (LS) and third author (DD) independently coded the first focus group responses and then compared for consistency. Any discrepancies in coding were discussed and resolved with the first author (NB). In this way, each author could critically challenge one another on differing perspectives and any potential biases.

A thematic framework was developed by generating major themes and subthemes in relation to the focus group questions and categorizing the associated responses iteratively. To maintain the context of focus group participant responses, they were listed under the questions from which they were derived and then categorized separately as a type of response. Throughout the coding process, regular meetings were held between the 3 authors to discuss and refine the thematic framework. Indexing was accomplished by coding each response in NVivo version 10 qualitative software (QSR International Pty Ltd, Burlington, MA, USA) with reliability checked by the second and third author through review of the NVivo file. Codes were considered saturated if more than 6 individuals supported the code, which is an appropriate number in qualitative data analysis [32]. At the final stage, the original responses were grouped according to the finalized themes and subthemes. Representative quotes were selected from the focus group responses to illustrate key themes and subthemes.

### Phase II: Plan

To address the needs identified in phase I, a plan was developed for the creation of the mobile phone app. First, a review of the most popular smoking cessation apps available on Google Play and the Apple Store was undertaken. Apps were assessed by their overall user ranking and number of downloads. The most popular and frequently downloaded cessation apps were then coded (Y or N) for feature content such as calculating days smoke-free, money saved, social support, health information tips and facts, quit planning, tracking performance and success, rewards, connection to quitline, and cost to purchase. Second, 2 sessions with experts were undertaken.

The first session was a mental mind mapping exercise that brought together a 5-person team of smoking cessation, behavior change, social media, and app development experts to design the content and the functions of CTC. This session lasted for 3 hours and was led by the team lead (NB). The session involved brainstorming with the objective of forming a shared understanding of the major facets that will be included in the app content to meet the needs of end users [33,34]. Experts were provided with the results of the review of mobile phone cessation apps by Abroms and colleagues [15], the findings from the assessment of the most popular and frequently downloaded cessation apps, and the summarized results of the focus groups with young adult smokers from phase I before engaging in the mind mapping exercise. The mind mapping exercise continued to evolve during the session until participants no longer had ideas to contribute and consensus was reached on the proposed content.

With the initial shared understanding of app content completed, the second session involved the design of the specific content and functionality of the app, which included 3 smoking cessation experts and 2 programmers. This session was facilitated by the team lead (NB) and employed a storyboarding design technique to carefully diagram the app content, user interface, and needed functionality [35].

### Phase III: Do

CTC was developed as a mobile hybrid and native app for the Android platform using the Web Informatics Development Environment technologies and toolkit [36]. Development of the app was done iteratively with the programming and research team and took 5 months. To seek feedback on the proposed features in the app from end users, 8 focus groups were conducted in Ottawa, Ontario, in a similar manner to phase I. Four focus groups were conducted in English and 4 in French. All focus groups included a mix of male and female participants. Fifty-seven participants were recruited by telephone via a panel of young adult smokers and were given an information letter and provided informed consent. Participants comprised 31 males and 26 females, and 20 with high school education or less, 16 with college diplomas or trade certificates, and 21 with university degrees. The age range was from 19 to 29 years. Thirty-one participants smoked 10 or more cigarettes per day and the remaining 25 smoked less than 10 per day, and one participant did not respond.

The sessions were guided by an interview schedule and focused on pilot testing of CTC. Each participant was given an Android smartphone with the app to try. Questions included asking what was liked and disliked about the app features and functionality, followed by questions on whether participants thought there was anything missing from the app. Focus groups lasted for 1 to 2 hours, and 2 researchers participated in each group. One researcher facilitated the session, and the other assisted and took notes. The thematic analysis of the focus group transcripts was conducted in the same manner as phase I.

### Phase IV: Act

This phase marked the launch of the CTC intervention, which was made available on Google Play Store and Apple iOS as of April 2013. The app’s final content and functions were modified according to the analysis of the feedback from focus groups in phase III. Phase IV provided the opportunity to track user behavior as a formative evaluation method over 1 year from July 2013 to June 2014. To assess app usage behavior, Google Analytics was implemented for Android users during the development of CTC. Usage statistics including number of users, age and gender of users, sessions, page views, average session duration, returning visitors, bounce rate (number of users who have left the app after only viewing the home page), and entrances and exits were monitored. For example, sessions refer to periods of time when users are actively engaged with the app, page views refer to the number of app pages that users look at, entrances are the number of times a user entered the app through a specific page, and bounce rate refers to the percentage of single-page visits. The data were quantitatively summarized to describe overall app usage. The usage data answered 2 key questions in terms of the formative evaluation: (1) how do users behave and interact with CTC? and (2) what content are users exposed to?

### Phase V: Study

This phase represents the outcome evaluation of an intervention. A rigorous evaluation of CTC was undertaken with the primary aim of determining its effectiveness for smoking cessation [23] (ClinicalTrials.gov NCT01983150). A parallel randomized controlled trial (RCT) with 2 arms was conducted in Canada with participants randomized to receive the CTC app (treatment) or an evidence-informed, self-help guide known as *On the Road to Quitting* (control) for a period of 6 months. The results of this phase will be presented in a subsequent publication.

## Results

### Phase I: Listen

Through the display of one of the most popular smoking cessation apps at the time (LiveStrong) and asking questions about cessation apps in general, young adults’ preferences regarding the content and features that they would like to see included in a smartphone app for smoking cessation were elicited. The focus group data resulted in 6 key themes: (1) positive reinforcement, (2) personalization, (3) social support, (4) quit support, (5) tracking the behavior, and (6) tracking quit benefits. Table 1 presents the major themes and associated subthemes, with representative quotes.

### Phase II: Plan

**Figure 1:**
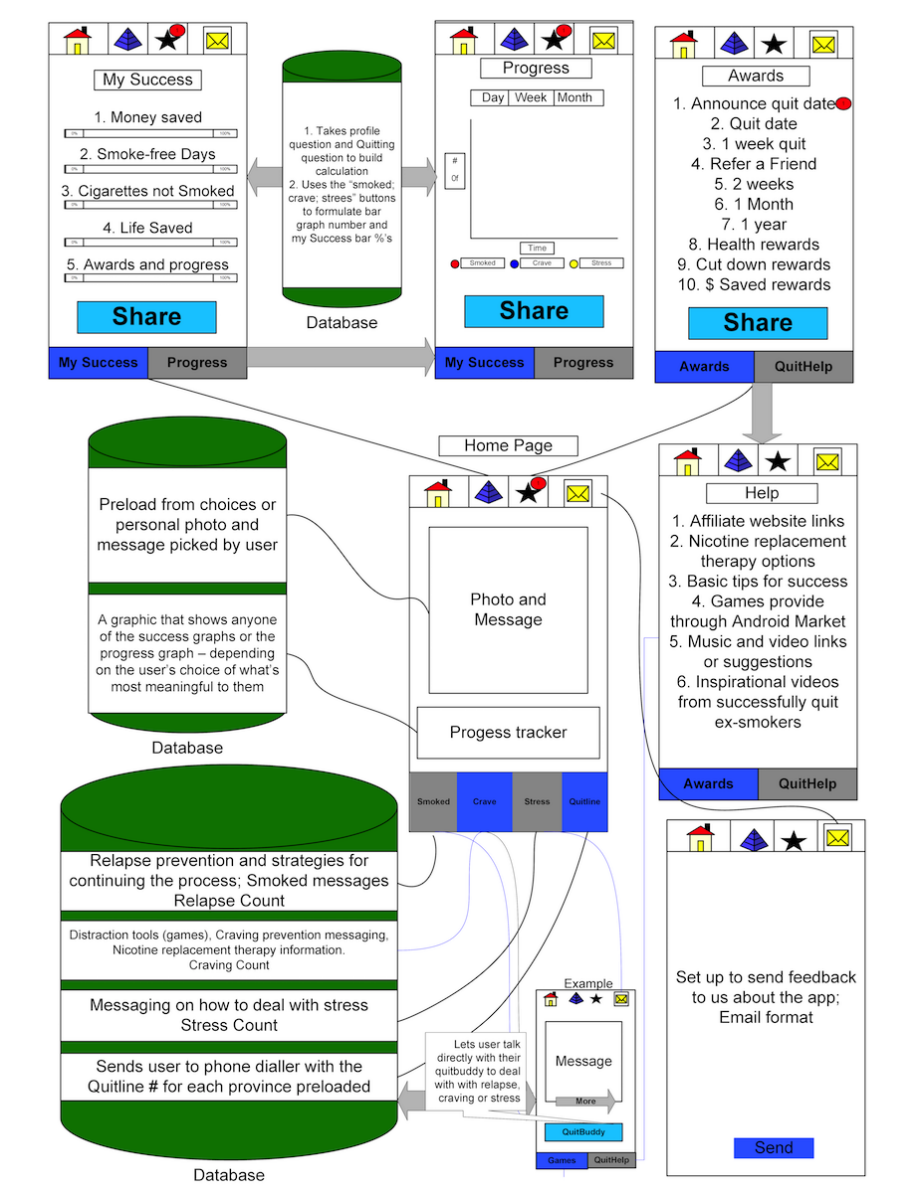
Phase II—example of storyboarding session results.

The 25 most popular rated (ranked 4.0 and higher out of 5) and downloaded apps (5000+ downloads) from Google Play and the Apple App Store as of January 2012 were assessed in terms of features. Fourteen of the apps included a calculation of days smoke-free, 13 provided money saved, 8 included social support or networking capability, 13 included health information tips and facts, 10 included a quit planning feature, 12 provided the ability to track performance and success, only 3 featured rewards for accomplishing goals, only 1 included the option to connect to a quitline, and 14 were free and of no cost to the user. Interestingly, 5 of the most popular apps promoted hypnosis as an approach to quitting smoking, an approach to quitting that is not empirically supported. Informed by the results of phase I and the cessation app feature assessment, mind mapping and storyboarding sessions were held with experts in smoking cessation and app programming. First, a mind map was generated that represented the ideas of experts for an evidence-informed cessation app. Second, the ideas were translated into app components and functionality using storyboards. Figure 1 is an example of a story board for CTC.

### Phase III: Do

Using a storyboarding technique with a group of experts and input from phase I focus groups, a prototype was developed on a whiteboard. Following this exercise, a digital prototype was developed and tested using smartphones with young adult smokers. Feedback was solicited from young adults regarding content and features, functionality, and whether anything was missing in the app that they would like to see. This stage also focused on usability of the app and user experience with the components to determine whether the app was perceived as helpful, motivating, and visually appealing. Findings from these focus groups were organized according to the app features and subfeatures, and then categorized as 2 types of user feedback: (1) user opinion and (2) suggested improvements. Table 2 presents representative quotes highlighting the major feedback from young adult app users.

**Table 1:** Phase I: Listen—User preferences for features and content in a smoking cessation smartphone app.

Theme and subtheme		Representative quote
**Encouragement**		
	Supportive messaging	“So have positive feedback and stuff like that. Just positive feedback, not negative.”
	Receiving awards	“...if you’re like a week without smoking or something, it comes up and tells you [that] you’ve done a week without smoking, good job...”
**Personalization**		
	Comprehensive profile setup	“Yes, I like that it has all the questions...It knows how old you are, it know how much you smoke, it knows what you want to keep track of, what you don’t, it knows how much money you’re spending on cigarettes on a daily basis. I like that.”
	Adding a personal touch	“Like [upload] a photo of your kid if you’re trying to quit for your kid.”
**Social support**		
	Social networking	“I like that idea...I’m not one to have everything on Facebook, but if it was something that I was proud of myself for, which would be quitting smoking, yeah, I’d like everyone to...acknowledge that.”
	Networking with other app users	“...maybe within the app have a network of everyone who is using the app and then that way anyone that you’re reaching out to is going to know exactly what you’re going through...”
	Quit buddy	“I think that’s good to have someone there that knows what’s going on.”
**Quit support**		
	Craving distractions	“I’d say, let’s say you get a craving right? You go into your phone and you whip out the app and you push a button and it gives you like a quick tip...like have a mint, have a sip of water something like that.”
	Immediate, live support	“I think that quick support thing is a good idea because if you’re talking with someone about your craving you’re probably not going to be having a cigarette while doing it.”
	Flexible quit approach	“The more options you can give the person who’s trying to quit, the better, whether they want to quit by themselves or quit today or quit 2 weeks from now or even a year from now. They should have that choice.”
**Tracking the behavior**		
	Identifying triggers	“I would use it to track...my cigarettes when I’m wanting to quit. Because I was looking for an efficient way to do that and I was actually carrying around a little pocket book for a while just so I could see. Because that’s where you have to start. That’s where I had to start anyway. So I definitely would use it in the planning stages to say like okay, I’m smoking now with who, what time and why.”
	Smoking frequency	“I don’t keep track of how many I smoke. I just assume, so if I was to keep track I’d probably be shocked. Yeah, this would be really helpful.”
**Tracking quit benefits**		
	Money saved	“Just because [money is] the most pressing on a day-to-day [basis]...You can see [money] coming out of your bank account on a daily basis...so it’s very easy to keep track of how much you would be saving...it’s an immediate thing.”
	Health benefit	“Yeah, the whole after 10 minutes of not smoking, you’re back to whatever [health], after 10 days of not smoking, back to this. Have a little timeline of what you’re doing so you can actually see the benefits of not smoking.”

**Table 2:** Phase III: Do—Thoughts and opinions from Crush the Crave (CTC) pilot test focus groups.

Feature and feedback type		Representative quote	
**Awards**		
	User opinion	“[It’s] fun and interactive. It’s almost like an old-school video game where it’s like, ‘You’ve unlocked this!’”	
		“I find it’s good because after so many days it takes time and it shows you. You see the savings of $1,000...and you see how much value there is and how much money you’ve saved.”	
	Suggested improvements	“Money is nice, and then you also show material things based on personal likes. You could have bought PlayStation 3. You saved enough to buy a PlayStation 3. If you didn’t smoke all these days, you could have bought that. Show them a picture of it, too. Again, people are visual. If you allow them to see, good.”	
		“So for people that are quitting for financial reasons it might be nice to be able to set up an alert at the end of the day saying “Way to go. You just saved ten bucks” so that I don’t have to maybe look at it. That little extra high five.”	
**Social supports through Facebook**		
	User opinion	“I know a lot of people who have quit and they always post on Facebook about it, and then they get their friends to support them. They’re like, oh me too, and they have conversations about it and talk over.”	
		“Facebook...It’s like, ‘Oh, first day of quitting. This sucks.’ And people are just like, ‘Oh, you’ll get through it, man.’ Just having your friends to support you while you’re doing that I think is helpful if you want that kind of connection.”	
	Suggested improvements	“If it’s more of an anonymous forum where you can just kind of say, ‘I’m really stressed out and smoking,’ as opposed to calling somebody up and talking to them, I might be inclined to use a forum...whereas Facebook, the quitter’s line or the call buddy, realistically, I’m never going to use that, so this would be an outlet perhaps that I might.”	
**Progress page to track smoking behavior and quit benefits**		
	User opinion	“It gives you a better idea of when you’re smoking most often and then you can figure out if you’re in those places hopefully not to smoke.”	
		“I find it’s good. A lot of people don’t understand why they smoke and in what situations and in the other screen it tells you, how do you feel and perhaps you don’t need to hang out with friends.”	
		“It gives a reminder, always in your face, so it really makes you realize, especially the saving money part. You could go out and buy a pack of smokes and then think nothing of it, but if it’s counting up how much money you’re spending, it’s like really eye opening.”	
	Suggested improvements	“...so now that I know my triggers are...and then what? If it continues to happen, what do I do? Just looking at it a couple of times a day is the same thing. A line graph would be a lot easier to read when you’re seeing your spikes. A bar graph...just shows volume...it’s like well, when during the day did you have 10 smokes? Did you have 10 smokes in 15 minutes, did you have 10 smokes in 15 hours?”	
**Quit help**		
	User opinion	“It goes directly to the games, I like that on my telephone. I can go on Facebook or Twitter [directly]. When I’m on Facebook or Twitter you think about something else. I think it’s a super good idea.”	
		“I also really liked how much information there was. The fact that it was in point form, it’s easy to read. It wasn’t long paragraphs, it was just the key points, and then if you wanted to learn more, you could go out on your own and look it up. I thought that was a good idea, because I wouldn’t read it if I saw a huge paragraph.”	
	Suggested improvements	“[Suggest] distractions in my area kind of thing, stuff to do, whether it be like go to the mall or the nature museum...Something other than I’m going to sit here with my phone and bugger around with my phone all day. Go get added distraction[s] from outside and stuff.”	
		“To have almost like a glossary page with all the different information and everything right there at my fingertips I think I’d be more likely to use that. Just in regards to the app itself it’s kind of the 1 page that is really jammed with stuff. Every other page is really, really simple.”	
		“It’s not that it was too much; it was that it was, like it comes at you like a jumble. You can organize or index it somehow.”	

**Figure 2:**
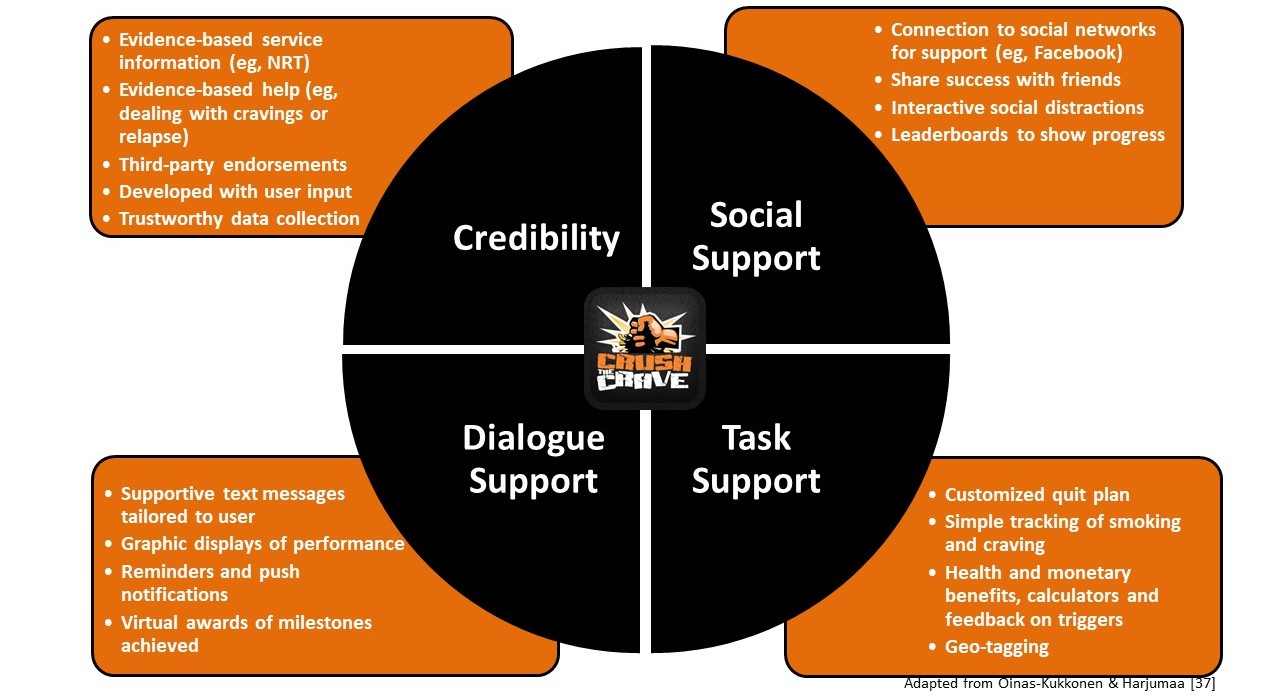
Evidence-informed design components of Crush the Crave (CTC).

### Phase IV: Act

**Figure 3:**
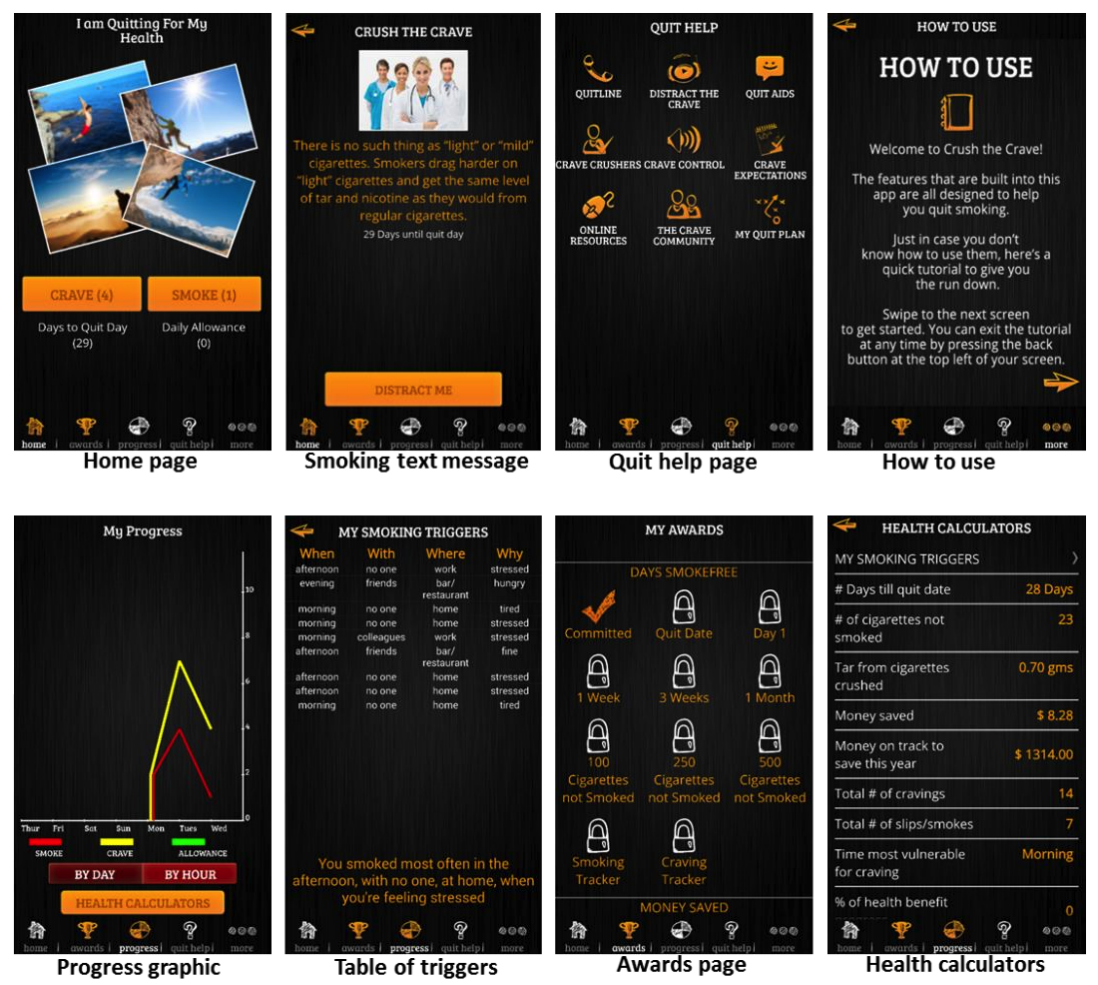
Sample screenshots of Crush the Crave (CTC).

Building on the findings from phase III, refinements were made to CTC. For example, the quit help information was beneficial but the organization was confusing. This was modified, and the information was rearranged in the future iteration of the app. Refinements also included the addition of a line graph to track progress rather than a bar graph. Furthermore, customization throughout the app was requested as a valuable and personal touch to CTC. For instance, enhancing personalization beyond a photo with the option to input affirmations or personal reasons for quitting and having user-specific smoking trigger situations in a database was indicated as helpful. Figure 2 details the ways in which the app content was organized using the persuasive technology framework by Oinas-Kukkonen and Harjumaa [37], which includes 4 key design components: credibility, task support, dialogue support, and social support. Figure 3 provides example screenshots of CTC. The app was launched on Google Play and iTunes on April 10, 2013.

In the 12 months from July 2013 to June 2014, there were 1987 Android users of CTC and 18,567 sessions, resulting in 59,384 page views or 3.2 pages per session. Users (n=1987) were 45.99% (914/1987) female and 60.99% (1212/1987) were between 18 and 34 years, 28.98% (576/1987) were 35 to 54 years, and 9.96% (198/1987) were older than 55 years. For sessions, 89.58% (1780/1987) were returning users, and 10.42% (207/1987) were new users (someone who had not previously registered with CTC). Overall session duration was 2:22 min on average. New users visited 6.4 pages per session with an average total visiting time of 4:23 min, whereas returning users visited 2.8 pages per session with an average total visiting time of 2:07 min per session.

#### User Behavior

The overall bounce rate was 58.6% (see Table 3) and ranged from 8.9% to 66.7% depending on the topic. For example, the bounce rate for the CTC home page indicates that 55.02% of users exited CTC from that page. In terms of user engagement, 59.39% (1180/1987) had between 9 and 200+ sessions with CTC. In addition, user engagement was strong with 89.58% (1780/1987) of users returning within the same day to use CTC. However, the majority, 70.99%, of these sessions had a duration or time on page of less than 10 seconds and 58.99% of these sessions involved only 1 page view, indicating opportunities for gaining additional insights into user behavior and improvements to CTC.

#### Content Exposure

Table 3 shows the pages viewed by users, with an overall average time per page of 1:04 min. The most viewed pages were the home page and quit help pages, followed by charting of progress toward reaching smoke-free goals and the message page associated with smoking. Entrances provide insight into the pages that serve as an entrance into using CTC, with the home page and progress pages serving that purpose more than others. Further, exits represent the last viewed page by users, and while this could indicate that users became frustrated or discouraged, it may indicate that users found what they are looking for. The craving distraction page and the helpful messages on encouraging users to quit smoking had the highest rates of exit (see Table 3).

### Phase V: Study

The results of phase V will be presented in an upcoming publication. The rigorous study of CTC using an RCT will add to the growing body of the evidence on the effectiveness of smartphone apps for smoking cessation. This evidence is necessary to move forward on decision making regarding the inclusion of technology-based mobile phone interventions as part of existing smoking cessation efforts made by policy makers and health care providers. Evidence from the trial will also inform the development of future apps, provide a deeper understanding of the factors that drive change in smoking behavior using an app, and improve the design of smoking cessation apps. The CTC trial is among the first to assess the effect of a comprehensive and evidence-informed mHealth smoking cessation app on a large sample of young adult smokers. Strengths of the trial include the high-quality research design and in-depth assessment of implementation. If effective, the trial has the potential to demonstrate that including mHealth technology as a population-based intervention strategy can cost-effectively reach a greater proportion of the population and help young adult smokers to quit.

**Table 3:** User behavior associated with Crush the Crave (CTC) app pages—July 2013 to June 2014.

Page	Topic	Page views	Unique Page views	Average time on page (min)	Entrances	Bounce rate (%)	Exits (%)
All pages	OVERALL	59,384	40,087	1:04	18,567	58.63	32.27
/homepage	Home page	13,165	8193	1:16	3,977	55.02	32.51
/quitHelppage	Quit help pages	5232	1219	0:31	383	8.88	10.07
/progressPage	Charting Progress	4770	3341	1:00	1176	53.23	26.21
/smoked	Smoke messaging	4153	2847	4:17	591	55.16	41.70
/morePage	More features	3971	2422	1:15	1114	62.48	32.21
/awardsPage	Awards received	3260	2328	0:49	919	66.70	32.09
/craved	Crave messaging	1927	1520	1:19	153	28.10	19.72
/locationPage	Location of smoking	1647	1154	0:04	124	—^a^	1.88
/triggersPage	Smoking triggers	1096	819	1:50	14	—	21.35
/distractMePage	Craving distractions	778	677	3:06	2	—	46.27
/quitlne	Quitline number page	131	118	1:26	1	—	14.5
/shareaward	Share an award on Facebook	32	19	0:13	3	—	28.12

^a^Data is not applicable.

## Discussion

### Principal Findings

In this paper, a step-by-step example of how evidence, theory, and user-driven feedback were incorporated into a smoking cessation app for young adults, CTC, is described. Development of this app was inspired by evidence that young adults should be a priority population for smoking cessation efforts [1,2], that young adults have saturated the mobile phone market [5], and that most available smoking cessation apps have been developed in isolation of theory and evidence [14,15,19,24]. The iterative process behind the development of CTC to address these gaps was made transparent through this paper. In doing this, leveraging smartphone technology for engaging young adult smokers may be enhanced [28,38,39].

To meet the needs of young adult smokers with a smartphone app, the design of the CTC was informed by multiple stakeholders at several points in time, including young adult smokers, tobacco control experts, social media experts, and researchers. This stakeholder input was combined with the USCPG [22] and principles of persuasive technology, which includes a systematic approach to behavior change using technology [21], to result in an evidence-informed app to help young adults quit smoking. In this regard, CTC included 4 key design components to meet young adults’ needs using persuasive technology: (1) credibility—ensuring that CTC was developed and backed by credible sources; (2) social support—providing opportunities to harness social support; (3) task support—providing young adults with practical support to help them with the task of quitting smoking; and (4) dialogue support—ensuring that young adults receive encouragement [37]. Findings from this formative evaluation demonstrate that CTC is a feasible and appealing option for helping young adults quit smoking, providing support for the development approach entailed. CTC is being used as intended with a high level of return visits and interaction with many of the key components of the app. It is noteworthy that the return visit rate was 89.58% (1780/1987) , which indicates that users were motivated to use CTC after downloading it.

A comprehensive look at how users respond to different aspects of the app was enabled through the use of both Google Analytics data and qualitative data via focus groups. Statistics on the uptake and use of the various app features and functions were provided through the analytics data, whereas user perceptions, usage, and contextual factors that might influence adoption and use were revealed through the qualitative data. This triangulation of data sources during formative evaluation of an mHealth intervention served an important role in shedding light on common themes and patterns of use that align with these common themes, as well as deviations. For example, whereas findings from focus groups were positive about the various aspects of the app, analytics data indicated low utilization of some features and functions, namely sharing awards via Facebook, using the quitline, tracking smoking location, documenting triggers, and using the craving distractions. Given that mHealth interventions operate in a real-world setting, it is of utmost importance to gather both types of data to cue attention to areas of strength, which may be enhanced, and areas of potential weakness, which may be further investigated and addressed [40]. Given the relative novelty of mobile platforms for health behavior interventions, efforts to understand user engagement via various measures is critical to the design of effective mHealth interventions [41].

In keeping with the STAR model approach [29], CTC is currently being evaluated in an RCT [23]. Young adult smokers were randomly allocated to CTC or the control group. Findings from this study will be used to inform intervention optimization by identifying aspects of the app that have the most potential to positively influence behavior change. Depending on the findings, CTC will be revised and subsequently tested.

### Limitations

There are several limitations of this study. First, the perceptions shared by phase I focus group participants were discussions of a hypothetical app. User perceptions reflected in the focus groups may change when an actual app is tested and used by the target population. Also, the perceptions shared by phase III focus group participants were discussions of an existing app, potentially constraining feedback to the existing functionality of the app. In addition, there may be differential preferences according to subgroups of young adults, particularly gender, and this should be an area for future research. Furthermore, group bias during focus groups may have kept alternative opinions from being voiced. In addition, although Apple iOS users likely reflected the same usage trends as Androids users, Apple iOS usage was not represented in the Google Analytics results and was limited in terms of descriptive statistics. Another limitation is the speed of technological changes and changing sophistication of users, which may limit the applicability of these findings for future app development. Furthermore, this rapidly changing context inherently requires constant refinement of the app. Finally, it must be noted that some segments of the young adult population may not own a smartphone and cannot be reached via smartphone interventions. However, this is changing, with smartphone ownership on the rise, and this might be a great way of closing a gap.

### Future Work

Although the results of this formative evaluation indicate that young adults have positively received CTC, engagement with the app and its related features and functions are critical to success [8-11]. Therefore, there is a need for more in-depth research on user engagement with the different components of the app and behavior change outcomes, and the results of the upcoming RCT will address this gap. In addition to this formal testing of the app, there is a need for additional qualitative research to shed light on some of the questions that remain about why certain features were more popular than others and what contextual factors influenced these trends. Given that mHealth interventions are situated in the contextually laden lives of end users, the value of qualitative research in mHealth is apparent.

The CTC app targets young adults. Given the existing knowledge gap in relation to mHealth intervention development for this population, efforts to standardize developmental practices are needed. Researchers are therefore encouraged to apply this development methodology to other app development projects to help refine and expand the development process of cessation apps.

### Conclusions

CTC is one of the first smoking cessation apps designed to meet the needs of young adult smokers. The development was informed by the inclusion of young adults in the design and the systematic application of multiple stakeholder input, scientific evidence, and theory. The STAR model approach proved to be a practical and comprehensive guide for development and evaluation, and it should facilitate optimization of mHealth interventions in the future.

## Abbreviations

CTC: Crush the Crave
mHealth: mobile health
RCT: randomized controlled trial
SMS: short message service
STAR: Spiral Technology Action Research
USCPG: US Clinical Practice Guidelines
